# Quantification of Cytokines in Lip Tissue from Infants Affected by Congenital Cleft Lip and Palate

**DOI:** 10.3390/children8020140

**Published:** 2021-02-12

**Authors:** Māra Pilmane, Nityanand Jain, Shivani Jain, Ilze Akota, Juta Kroiča

**Affiliations:** 1 Department of Morphology, Institute of Anatomy and Anthropology, Riga Stradiņš University, LV-1007 Riga, Latvia; mara.pilmane@rsu.lv; 2 Department of Oral and Maxillofacial Surgery, Genesis Institute of Dental Sciences & Research, Ferozepur, Punjab 152002, India; drvanijain@rediffmail.com; 3 Department of Oral and Maxillofacial Surgery, Faculty of Dentistry, Institute of Stomatology, Riga Stradiņš University, Dzirciema Street 16, LV-1007 Riga, Latvia; ilze.akota@rsu.lv; 4 Department of Biology and Microbiology, Faculty of Medicine, Riga Stradiņš University, Dzirciema Street 16, LV-1007 Riga, Latvia; juta.kroica@rsu.lv

**Keywords:** cleft lip, cytokines, ELISA, cleft palate, interleukin, newborn

## Abstract

Cleft lip and palate are amongst the most common congenital malformations worldwide presenting with variable manifestations. Previous research has been primarily focused on the genetical aspects of its complex and multifactorial etiology. In the present study, we investigated the role of cytokines as mediators of epithelial–mesenchymal crosstalk and local site inflammation in cleft affected infants. Lip material was obtained from 12 children aged before primary dentition who suffered from orofacial clefting. The quantification of 12 cytokines (Interleukin-2,4,5,6,10,12,13,17A, Tumor Necrosis Factor-α, Interferon-γ, Transforming Growth Factor beta-1 and Granulocyte-Colony Stimulating Factor) was done using ELISA. Nonparametric Spearman Rho was used to ascertain the correlation between the expression levels of different cytokines. A significantly strong positive correlation was found between IL-2 and IFN-γ coupled with an IL4/IFN-γ ratio favoring IFN-γ. These findings indicate a shift towards the preferential activation of the Th1 differentiation pathway. Further, a pathological reduction in TGFβ-1 levels was noted, which may contribute to mucosal damage. IL-6 was more highly correlated to IFN-γ and IL-12 indicating its potential proinflammatory role in cleft affected tissues. This preferential activation of Th1 cell differentiation and consistent expression of IL-2,6,13 and TNF-α in cleft patients may indicate certain underlying mechanisms for inflammation mediation at the site of clefting.

## 1. Introduction

Orofacial defects such as cleft lip and/or palate are one of the most common congenital malformations worldwide with an estimated prevalence rate of 1.7 to 2.0 per 1000 live births [1,2,3]. A cleft, defined as an abnormal space or gap in the upper lip, alveolus, and/or palate [4], can be unilateral or bilateral [5]. The malformations can manifest with variable severity and range from occult to overt presentation with etiology ranging from syndromic/genetic to teratogenic/environmental factors [6]. The malformations in almost all cases require orthognathic surgical intervention for both functional and aesthetic purposes [7,8]. This surgical intervention (currently the gold standard) parallelly also translates to a huge socioeconomic burden on the healthcare system with estimates exceeding $100 million every year in US alone [6,9]. The surgical care is often followed by multiple psychologically exhaustive sessions extending from childhood to adolescence to address the associated problems of speech, dental occlusion, fluid buildup in the ears, and maxillary growth deficiency [6,10].

The development of the face and oral cavity is a highly complex and orchestrated process involving the development and maturation of multiple tissues that must merge and fuse under appropriate conditions and timing [11]. The three most crucial events in the process migration of cell masses, the fusion of facial processes and the subsequent differentiation of tissues [12] are tightly regulated by various genetic and environmental factors. Disruption in any of the involved genes and/or inhibition of cell functions by environmental factors (like teratogens) will lead to clefting [11]. This makes clefting a rather complex and multifactorial disorder resulting due to the accumulation of several biochemical and development events [13]. Whilst the genetic side has evolved considerably [14,15], the other side remains to be investigated and elucidated.

During facial development, the facial primordia consisting mainly of migrated neural crest cells are formed [16]. These crest cells with ectoderm form the maxillary processes which gives rise to palatal shelves around the 45th embryonic day. Later, an intrinsic elevating force (due to the accumulation and hydration of hyaluronic acid-1 secreted by palatal mesenchymal cells) overcomes the resisting forces from the tongue, causing the palatal shelves to elevate horizontally above the dorsum of the tongue. In the midline, the epithelia of the approximating shelves fuse with each other using desmosomes and cell adhesion molecules to form the midline epithelial seam [16].

The fusion of shelves leads to palatal expansion in height by the thinning of the seam, followed by migration of epithelial cells to the nasal and oral aspects of the palate [16]. The seam then degenerates to establish mesenchymal continuity across the horizontal plate. This programmed cell death of the epithelium is shown to be initiated by the underlying mesenchyme [16]. This epithelium–mesenchymal crosstalk is mediated by extracellular matrix molecules (like collagen molecules), through soluble factors (cytokines, growth factors), direct cell–to–cell contact (paracrine, autocrine), or a combination of all of the above mediators [16].

Deviations, aberrations, and complications in these orchestrated sequelae of events leading up to and/or during the fusion of the shelves will eventually lead to clefting of the palate with varying severity [16,17,18]. For cleft lip, the critical/ defining event seems to be the contact and fusion of the medial and lateral nasal processes [12]. Hence, it becomes essential to understand and evaluate the role of signaling molecules such as cytokines in the pathogenesis of cleft lip and palate that can aid in better prediction, diagnosis, and treatment of the defects.

Cytokines are small, soluble, nonstructural proteins with low molecular weight having complex regulatory influence on inflammation and immunity [19]. They are intercellular messengers in the immune system that integrate functions of several cell types in various body compartments to form a coherent immune system [19]. They are involved in growth, differentiation, activation, cell migration, fusion, and apoptotic functions, all of which contribute towards the complexity of the tissue organization in the craniofacial region [6,20]. Cytokines can be grouped into three broad groups based on the nature of their responses, which can then be subgrouped based on the receptors used for signaling [21].

Group I consists of cytokines involved in adaptive immunity and produced primarily by T-lymphocytes that have recognized an antigen specific to that cell. Interleukin (IL)—2,4,5 and 13 are some major members of this group. Group II consists of cytokines involved in proinflammatory signaling like IL-6 and 17A, tumor necrosis factor (TNF)-α and Interferon (IFN)-γ. Finally, Group III consists of cytokines involved in anti-inflammatory signaling like IL-10 and 12. Other major cytokines include transforming growth factor (TGF) -β which inhibits T and B cell proliferation and hematopoiesis whilst promoting wound healing. Granulocyte-colony stimulating factor (G-CSF), another key cytokine, stimulates the bone marrow to produce granulocytes and stem cells.

Over the years, many techniques have been developed and used for the quantification of cytokine levels in tissue samples like ELISA (enzyme linked immuno-sorbent assay), multiplex arrays, Western blotting, and radioimmunoassay, with the former two being the most used. Whilst the basic working concept behind all the techniques more or less remains the same, i.e., immunorecognition of specific antigen by antibody, the main difference lies in the procedure methodology, associated costs, and specificities. ELISA remains the gold standard due to its accurate and sensitive detection of cytokine levels along with being highly quantitative and generally reproducible [22]. Multiplex arrays offer the added benefits of evaluating the complexity and dynamic nature of cytokine-mediated inflammatory responses along with offering substantial cost and sample savings over traditional ELISA measurements [22]. However, the main concern with such multiplex arrays is the requirement of ascertaining the nonreactivity of individual cytokines to all other antibodies, coupled with limited industrial expertise [22], thereby restricting them from being the next gold standard in cytokine quantification.

In the past, studies have tried to quantify the levels of various cytokines in cleft patients [5,23,24]. However, two of the studies used blood samples for quantification [23,24] while the third study tested the lip tissue for only six cytokines [5], leaving a huge gap in analyzing and understanding the levels of cytokines in cleft tissue and their potential role in pathogenesis and tissue remodeling. The present study, therefore, aims to fulfill this gap and contribute towards our present understanding of the concentrations of different cytokines in cleft lip and palate affected tissue and regarding their possible role in these tissues.

## 2. Materials and Methods

### 2.1. Profile of Study Participants

The present study included lip material obtained from 12 pediatric patients who reported in the Department of Oral and Maxillofacial Surgery, Institute of Stomatology, Riga Stradiņš University (RSU), Latvia. The protocol and workflow for the present study was approved and consented by the Research Ethics Committee (REC) of Riga Stradiņš University wide approval No. 5/25.06.2018. All participants included in the study gave written as well as oral informed consent (provided by the parents) in the language of their understanding (English, Latvian and/or Russian) for participation in the study. The study protocol was designed in accordance with the Helsinki Declaration, 1975 (as revised in 2008).

The infants were aged between 3 to 13 months at the time of tissue collection and were indicated for plastic surgery for either bilateral or unilateral clefts. None of the infants had erupted primary dentition and were not previously diagnosed with coexisting genetic syndromes, chromosomal abnormalities, or immune deficiencies. As shown in Table 1, four infants were born preterm whilst two were born post-term. One infant demonstrated multiple stigma simultaneous to cleft lip and palate. Additionally, the mothers of five infants were reported to have undergone medical treatment (undisclosed) during their pregnancy while one infant showed syndromic cleft. Only three infants were born without any known teratogenic influence during the maternal pregnancy course.

### 2.2. Data and Sample Collection

Tissue samples were collected from the cleft lip immediately after surgery and were transported under ambient conditions in a coolbox to the Department of Biology and Microbiology, RSU where they were cryopreserved at −80 °C in 1 × PBS (Phosphate Buffered Saline). Upon receipt and appropriate registration, the tissue samples were transported to Department of Morphology, Institute of Anatomy and Anthropology, RSU, under ambient conditions for further analysis. The samples were assigned randomized sequence tags and their identity was not disclosed at any time to the researchers and/or lab assistants in accordance with the protocol. Only patient history (as shown in Table 1) was kept with the sequence tags.

### 2.3. Sample Preparation and ELISA

The patient tissue samples were then finely sectioned into 1–2 mm pieces using microtome and homogenized in 1× PBS buffer using the Laboratory Line Polytron PT1200E homogenizer (Kinematica AG, Malters, Switzerland). RIPA (Radio-immuno-precipitation assay) buffer (ThermoFisher Scientific, Waltham, MA, USA) supplemented with protease inhibitors (Sigma-aldrich, St. Louis, MO, USA) was added to the samples and left at room temperature for 30 min and vortexed several times. The samples then were centrifuged at 14,000 rpm (revolutions per minute) at 4 °C for 15 min to remove lysed cell particles. The resulting supernatant was collected and frozen at −80 °C. ELISA was performed using the Multianalyte ELISArray Kits (Qiagen) in accordance with the manufacturer’s guidelines.

### 2.4. Cytokines Studied and Statistical Analysis

The samples were investigated for the concentrations of 12 cytokines—IL-2, IL-4, IL-5, IL-6, IL-10, IL-12, IL-13, IL-17A, TNF-α, IFN-γ, TGFβ-1 and G-CSF. The data were stored and analyzed using MS Excel (MS Office 365) and SPSS v26.0 (IBM Corp., Armonk, NY, USA). Statistical significance was set at *p* < 0.05. Quantitative variables were presented as mean (±standard error). Spearman Rho was used for correlation analysis between cytokines.

## 3. Results

### 3.1. Mean Concentration of Cytokines in Patients

All cytokines investigated were detected by ELISA in the lip tissue sample (except for IL-5) as shown in Table 2. The highest concentrations were recorded for TGFβ-1 followed by TNF-α. The lowest concentrations were recorded for IL-17A followed by IL-2. Coefficient of Variation (CV%) was calculated to analyze the inter-sample variations. A large variation was observed in the concentrations of IL-2, IL-17A, IFN-γ, TNF-α and G-CSF (all having CV > 50%). On the other hand, IL-4, IL-12, and IL-13 showed less variance amongst the samples (all having CV < 30%).

### 3.2. Concentration of Cytokines in Individual Patients

A complete patient profile and clinical diagnosis is shown in Materials and Methods (Section 2.1). Based on the clinical diagnosis of the patients, they were divided into 4 groups for comparative evaluation of cytokine levels. Group A comprised patients diagnosed with Cheiloschisis; Group B comprised patients diagnosed with Cheilognathoschisis; Group C had patients with Cheilognathouranoschisis, and Group D had patients with Uranoschisis (Table 3 and Table 4).

### 3.3. Correlation between Cytokines

A correlation matrix (Table 5) was analyzed to ascertain the relationship between the different cytokines investigated in the present study. A very strong and significant positive association was noted for IL-2 with IL-17A, IFN-γ and G-CSF (ρ > 0.80; *p* < 0.01). IL-4 was found to be strongly correlated with IL-10 and IL-12. Interestingly, a perfect positive correlation (ρ = 1.000; *p* < 0.01) was observed for IL-4 with IL-17A, IFN-γ and TNF-α. IL-17A also showed a perfect positive correlation with IL-10 and IL-12. A similar relationship was demonstrated by IL-10 and IL-12. A weak nonsignificant negative correlation was found between TNF-α and IL-17A and TGFβ-1. Furthermore, TGFβ-1 and IL-10 demonstrated a perfectly negative relationship, although it was statistically not significant (*p* > 0.05).

## 4. Discussion

Over the years, cleft lip and palate have been a major recipient of research dealing with developmental causes and mechanisms in comparison with most other congenital malformations [25]. This is partially because of its high incidence rate when compared with other congenital malformations and partially because of its multifactorial etiology, which has not yet been understood completely. A blend of genetical, experimental and epidemiological studies have been undertaken that have furthered our understanding of the underlying processes while generating a variety of hypothesis that could cause orofacial clefting. Whilst detailed observations are available that describe multiple events, ranging from palate shelf horizontalization to the critical phases of closure, there is still a need to account for external factors (like in vivo vs. in vitro experimental differences, intra- and interspecies polymorphisms etc.) before specific cause-and-effect mechanisms can be isolated [25].

Various embryologic events have been identified by researchers that could lead to the clefting of different orofacial structures. The identification of these events has in turn led to postulation of various mechanisms for such events. A major mechanism being investigated and gaining traction is the role of cytokines (or growth factors) in the mediation of crosstalk between epithelial and mesenchymal cells. This role is especially crucial to understand and elucidate during the fusion phase of palatogenesis, which requires the coordinated apoptosis of epithelium whilst the processes on the two sides fuse in the midline. Further, the presence of innate lymphoid cells (ILCs) reported at the vermillion in neonates and young children undergoing cleft lip reconstruction showed the role the cytokines play in lowering the surgical site infection rates in cleft lip reconstruction [5]. This finding bolstered and proved the pivotal role of ILCs (and in turn cytokines) in establishing a desirable microenvironment during and after tissue development under healthy and normal status [5].

In the present study, we therefore aimed to identify and quantify various cytokines in the lip tissue of the cleft that affected neonates and young children to ascertain and further our understanding of the varied roles of cytokines. In all our patients we observed that IL-2,6,13, and TNF-alpha were always present (except IL-6 in patient 6) and detectable in the cleft lip affected tissue. IL-2, a member of the IL-2–related family, stimulates T-cell growth through the IL-2 receptor. It exhibits myriad effects on the immune system in addition to promoting CD4 and CD8 T-cell proliferation and differentiation into effector T cells and memory T cells [26]. Recently, IL-2 has been shown to play an important role in inducing oral mucosa death in human oral squamous cell line [27]. The authors demonstrated that IL-2 treatment induces inflammatory response and oxidative stress via upregulation of the NF-κB pathway in oral squamous cells [27].

Further, it has been described that the ratio of “inhibitory” vs. “activating” Th-1 cells at the site of inflammation may help in distinguishing whether an immune response will perpetuate or will be contained and eventually shut down [28]. An IL-2:IL-10 ratio favoring IL-2 will indicate antigen-induced proliferation of T cells while a ratio favoring IL-10 will only partially inhibit the T cell proliferation [28]. Similarly, an IFN-γ:IL-10 ratio favoring IFN-γ indicates enhanced LPS (lipopolysaccharides)-induced monocyte activation (as assessed by TNF-α production) while in the reverse case, IL-10 antagonized the effects of IFN-γ [28]. It has also been demonstrated that IL-2 treated natural killer cells lysed a significantly higher proportion of mesenchymal stem cells and dental pulp stem cells, which were supplemented with higher IFN-γ levels in cocultures containing both stem cells and IL-2 treated NK cells [29]. Taken together, it is clear that both IL-2 and IFN-γ are highly correlated in promoting mesenchymal cell death. Indeed, our analysis in cleft lip and palate tissues showed significantly high positive correlation between the two cytokines.

Amongst the various postulated causes of clefting of primary palate, initial mesenchymal deficiency, delayed ossification, decreased premaxilla volume, increased apoptosis, or increased bone resorption due to a lack of functional forces on the primary palate are considered the most important [30,31,32]. It is unclear as of now, whether IL2-IFN-γ-IL10 axis could play a pivotal role in initial mesenchymal cell death and the increased apoptosis of epithelial cells during embryonic development, but it is a potential direction for further investigations. In most of our samples, IL-10 levels remained below the levels of the negative control (undetectable factor) and hence, it leads us to hypothesize that perhaps IL-2-IFN-γ may be an important mediator of apoptotic signals due to the low levels of IL-10 secretion in cleft patients.

IL-4 and IL-13 are the hallmark cytokines of Type II inflammatory response and are secreted by CD4+ T cells, basophils, eosinophils, mast cells, and NK T cells, along with appropriately stimulated ILC-2 cells [33,34,35,36]. IL-4 is the main cytokine in Th2 cell-mediated immunity protecting the cells from extracellular pathogens. If an immune reaction favors IL-4 release, it is correlated with high IL-10 and IL-5 release as also seen in our results. However, since IL-5 was not detected in any sample and limited IL-10 detection, it is no surprise that IL-4 also remained undetected in various samples. The IL-4:IFN-γ ratio can determine whether the immune reaction is favoring Th2 or Th1-mediated immunity, respectively [37]. Yet again, our results support that the IFN-γ-mediated immune reaction tends to be more favored in cleft affected patients. Further, it has been demonstrated that a shift from Th1 to Th2 response is an essential requirement for a successful pregnancy since IFN-γ can be harmful to the placenta and fetus [38,39].

Additionally, it has been shown that initial priming of immune system against allergens occurs in the prenatal period as evidenced by the detection of proliferative responses to allergens in cord blood mononuclear cells [40,41]. Hence, the maternal influence on cytokine levels could not be ignored. The maternal influence could be allergen-specific due to leakage of allergens, anti-idiotypic antibodies or allergen–antibody transfer across the placenta [42] or nonspecific influence through the cytokine environment at the materno–fetal interface. Postnatally as well, levels could be influenced due to cytokine delivery via breast milk [43,44]. This could partially explain the reverse trend of cytokine ratios observed in some of our patients, although more complex interactions still need to be explored. Further, whether the immune priming and maternal influence on fetal immune system occurs before or after palate closure (or nasal closure) could also help to further understand the pathogenesis. An interesting observation was, however, reported by Perrier et al., whereby they found that IL-4 might play a proinflammatory role in oral mucosal epithelial cells [45]. They found that IL-4 increased IL-1α (potent proinflammatory cytokine) production without affecting the IL-1 receptor antagonist expression [45]. Whether this is true for cleft patients as well needs to be investigated in future studies.

TGFβ-1 is a multifunctional cytokine that regulates several events in many cell types. It is a strong inhibitor of cell growth and a differentiation factor in various epithelial cells including oral mucosal epithelial cells and skin keratinocytes [46,47,48]. It is known to be a strong inducer of inflammation by upregulation of IL-1α in skin keratinocytes [48]. However, in the same study by Perrier et al., it was found that in oral mucosal epithelial cells, TGFβ-1 favored the production of IL-1 receptor antagonist by more than 100-fold, leading to inactivation of IL-1 bioactivity. In other words, it promoted an anti-inflammatory response [45]. Since no controls could be analyzed for the present study, a reference range for TGFβ-1 from the study by Okamoto et al. was considered for comparison [49]. They reported TGFβ-1 serum concentration of 61.7 ± 18.5 ng/mL or 61,700 pg/mL in children aged 1–14 years which is significantly higher than the levels detected in our samples [49]. It has been reported that a pathological decrease in TGFβ-1 (along with decreased IL-1 receptor antagonist) levels may contribute to mucosal damages such as oral erosions associated with infections as also seen in Sjögren Syndrome [45,50].

IL-12 is another potent Th1 immune response system cytokine that is produced by antigen presenting cells (APC), dendritic cells (DC) and monocytes/macrophages, either after stimulation with bacterial cell wall products or after direct interaction with T cells via CD40/CD154 (CD40L) [37]. The latter pathway is more complex and tightly regulated by other cytokines like IL-4 and IFN-γ, which are known to upregulate bioactive IL-12, as is also evident in our results [51,52]. IL-12 has been further shown to induce IFN-γ production during the primary stimulation of Th naïve cells [53,54]. IL-5 is a cytokine primarily produced by the Th2 cells and mast cells when the cells are stimulated with IL-2 and certain other agents [55,56]. The autocrine production of IL-5 by eosinophils has also been demonstrated that could take part in pathogenesis of chronic inflammatory conditions [57]. It promotes eosinophil differentiation, migration, activation, degranulation, and the survival of eosinophils along with being an essential cytokine for eosinophil maturation [55]. IL-5 remained undetected in all samples and our results were in concordance with other studies where they evaluated serum concentrations of IL-5 in children [58].

In mice models, it has been shown that although TGFβ inhibits both Th1 and Th2 immune systems, it preferentially inhibits Th2 differentiation leading to reduced expression of IL-4, 5 and 13, while allowing normal induction of *Ifng* gene (encoding IFN-γ) [59]. This could partially explain the non-detection of IL-4 and IL-5 in the majority of the samples since the samples with relatively high TGFβ reported non-detectable levels of IL-4.

IL-6 is a multifunctional cytokine involved in multiple processes such as the regulation of the immune response, hematopoiesis, inflammation, cell survival, apoptosis, cell proliferation and oncogenesis [60,61]. What makes IL-6 rather intriguing is its dual nature in inflammatory processes i.e., as an anti-inflammatory (via the classical pathway) and as a proinflammatory (via the trans-signaling pathway) cytokine [62]. IL-6 can induce the secretion of acute phase proteins, can upregulate neutrophil recruitment and expression of cell adhesive molecules and can switch from neutrophil to macrophage-induced inflammation along with IL-1 and TNF-α [62]. On the other hand, it can stimulate T cell proliferation and, together with IL-4, participates in the generation of the Th2 immune response [62,63]. It has been shown that IL-6 is produced by human mesenchymal stem cells either after induction with TNF-α, IL-1b and IFN-γ or spontaneously [64,65]. It has also been shown to induce the production of IL-10 by different types of cells [63]. In our study, IL-6 was more highly correlated to IFN-γ and IL-12 than IL-4, indicating its potential proinflammatory role in cleft affected tissues.

IL-17A, yet another multifaceted cytokine, is secreted by Th17 cells that can be induced in the presence of TGF-β/IL-1β, IL-6 and IL-23 [66,67]. It can induce epithelial secretions of granulopoietic factors like G-CSF, which stimulates neutrophils [68], as shown by thr significant positive high correlation seen in our results. Alone, it often induces a weak response, but it may synergize with other cytokines, like TNF-α, to enhance and prolong proinflammatory responses [69,70]. It is evident from the literature that, in normal pregnancy, materno–fetal interactions regulate the levels of various cytokines in such a way that preferential activation of the Th2 pathway is maintained against Th1 pathway activation [71]. Preferential activation of the Th1 pathway (upregulation of IL-2, TNF-α and TGFβ-1) may lead to abnormal placental and embryonic development with serious imbalances leading to fetal death [71].

Previous pieces of research in mice models have depicted that modulated cytokine expression directly impacts its sensitivity to environmental teratogens [72]. TGFβ family cytokines and receptors, for example, have been shown to be involved in glucocorticoid-induced cleft lip [72]. Additionally, it has been demonstrated that maternal metabolism plays a crucial role in determining the response of the embryo to environmental teratogens. TNF-α, TGFβ, IL-2, IL-6 and other cytokines have been shown to induce cytochrome P450 isoenzyme expression, thereby modulating the response to teratogens [71,73]. Maternal and paternal exposures to smoking, alcohol, vitamin use etc. have also been shown to increase the risk of clefting via gene–environment–cytokine interactions [74,75]. Finally, certain maternal hormones have also been implicated in modulating cytokine expression in embryo. IL-4 and 5 are for example, decreased by dihydrotestosterone [76] while glucocorticoids decrease IL-2 and IFN-γ [77]. Progesterone, an abundant hormone in pregnancy, especially at materno–fetal interface, promotes Th2 pathway activation [71,78]. Relaxin, a polypeptide hormone, on the other hand counterbalances the effects of progesterone [79]. Estrogen also plays a role in cytokine expression modulation [80]. Whether early detection of the fluctuations of these hormones in the mother’s serum can serve as markers of clefting needs to be investigated in future studies.

It is clear from the above presented discussion that the cytokines play crucial yet varied roles in the development, pathogenesis, and manifestations of cleft pathologies. The present study emphasizes the need for more intensive and broader studies regarding the role of cytokines in immunology and their roles as mediators of cross-talk so as to develop and augment patient-specific diagnosis, treatment, and patient management modalities. Immune signaling and regulation consists of a complex web of interactions that is yet to be fully understood and is under constant revision. The present study has some limitations like lack of control samples and the low number of samples investigated. However, the availability of control lip samples from normal newborn is extremely difficult due to parental concerns and the tender age of the child. Moving forward, we encourage more studies to be undertaken that might perhaps investigate different cytokines like IL-1, 8 etc. in a bid to further our understanding of cleft pathologies.

## 5. Conclusions

The complex and intertangled interactions between various cytokines, along with varied dual roles of certain cytokines (IL-4 and 6), can play a pivotal role in the pathogenesis and manifestation of orofacial clefts. The consistent expression of IL-2,6,13 and TNF-α in the cleft lip and palate affected tissue indicates the role of these cytokines in the pathogenesis of these anomalies. The apparent downregulation of anti-inflammatory cytokines like IL-4 and IL-10 coupled with pathological decrease in TGFβ-1 may indicate potential interactions that may contribute to clefting and the subsequent local site inflammation.

## Figures and Tables

**Table 1:** Profile of study participants and clinical diagnosis.

Patient No	Age (in Months)	Gender	Clinical Diagnosis *	Remarks
1	7	M	Cheiloschisis partialis sinistra	Preterm birth in gestational week 37; Rh conflict; mother showed glucose tolerance disturbance
2	6	F	Cheilognathoschisis dextra	Born in the gestational 42nd week; cleft detected in ultrasound in week 22; history of cleft lip and palate in family tree
3	4	M	Cheilognathouranoschisis dextra	Born in the gestational 41st week; cleft detected in the ultrasound in week 22; mother reported urinary tract infection during the pregnancy
4	10	F	Cheiloschisis partialis dextra	Preterm birth in gestational week 39
5	13	F	Cheilognathoschisis bilateralis	Cleft detected in the ultrasound in week 28; mother used Paracetamol during the pregnancy due to tooth aches
6	4	M	Cheilognathouranoschisis bilateralis	Preterm birth in gestational week 39; both parents were regular smokers
7	4	M	Cheilognathoschisis sinistra	Multiple stigma: dysplastic ears, palm stigma
8	4	M	Uranoschisis partialis sinistra	-
9	7	M	Cheiloschisis partialis sinistra	There was reported high risk of abortion during pregnancy
10	4	M	Cheilognathouranoschisis sinistra	-
11	4	F	Cheilognathouranoschisis dextra	-
12	4	M	Cheiloschisis dextra	Preterm birth in gestational week 39

* Clinical Diagnosis is provided in Latin; Cheiloschisis—Cleft lip; Cheilognathoschisis—Cleft lip and alveolar ridge; Cheilognathouranoschisis—Cleft lip, alveolar ridge and palate; Uranoschisis—Cleft palate; sinistra—left; dextra—right; partialis—partial; bilateralis—bilateral.

**Table 2:** Mean concentration and Coefficient of Variation (CV%) of the cytokines in pg/mL.

Cytokine	Mean Concentration	Standard Deviation	Minimum Concentration	Maximum Concentration	Coefficient of Variance (CV%) *
IL-2	1.58	1.20	0.19	4.29	75.95
IL-4	1.06	0.28	0.78	1.40	26.42
IL-5	-	-	-	-	-
IL-6	0.59	0.18	0.32	0.87	30.51
IL-10	1.13	0.47	0.72	1.96	41.59
IL-12	0.93	0.25	0.65	1.25	26.88
IL-13	0.57	0.17	0.26	0.99	29.82
IL-17A	1.11	0.74	0.10	2.48	66.67
IFN-γ	0.79	0.50	0.23	1.66	63.29
TNF-α	36.93	20.84	14.69	88.68	56.43
G-CSF	0.70	0.47	0.11	1.70	67.14
TGFβ-1	152.01	69.83	90.30	248.40	45.94

* CV% is defined as the ratio of standard deviation and mean; IL-5 could not be detected in any of the samples since the concentration of IL-5 in the samples was below the negative control (undetectable factor).

**Table 3:** Concentration of interleukins in individual patients measured in pg/mL.

Patient No	IL-2	IL-4	IL-6	IL-10	IL-12	IL-13	IL-17A
**Cheiloschisis**							
1	0.185	0.776	0.361	0.723	0.739	0.412	0.100
4	0.277	0.790	0.315	0.722	0.648	0.453	-
9	1.650	-	0.608	-	-	0.565	1.305
12	1.618	-	0.516	-	-	0.501	1.011
**Cheilognathoschisis**							
2	0.798	1.401	0.678	1.290	1.248	0.576	0.777
5	0.554	1.248	0.867	1.150	1.118	0.615	0.660
7	1.715	-	0.573	-	-	0.600	0.939
**Cheilognathouranoschisis**							
3	0.839	1.104	0.428	0.945	0.912	0.605	0.573
6	4.292	-	-	1.960	-	0.261	-
10	2.435	-	0.620	-	-	0.583	2.480
11	2.912	-	0.868	-	-	0.992	2.248
**Uranoschisis**							
8	1.684	-	0.608	-	-	0.643	0.974

Clinical Diagnosis is provided in Latin; Cheiloschisis—Cleft lip; Cheilognathoschisis—Cleft lip and alveolar ridge; Cheilognathouranoschisis—Cleft lip, alveolar ridge, and palate; Uranoschisis—Cleft palate; “-” indicates concentration of interleukin in the sample was below the negative control (undetectable factor).

**Table 4:** Concentration of other cytokines in individual patients measured in pg/mL.

Patient No	IFN-γ	TNF-α	G-CSF	TGFβ-1
**Cheiloschisis**				
1	0.230	28.935	0.199	*
4	0.300	30.264	0.108	*
9	0.768	25.838	0.755	248.400
12	0.757	19.947	0.582	120.224
**Cheilognathoschisis**				
2	-	88.683	0.333	90.798
5	0.443	55.631	0.758	*
7	0.758	14.816	0.696	100.920
**Cheilognathouranoschisis**				
3	0.369	39.422	0.441	*
6	-	51.098	-	90.300
10	1.585	32.550	1.695	215.720
11	1.664	41.300	1.320	120.224
**Uranoschisis**				
8	0.982	14.687	0.794	109.402

Clinical Diagnosis is provided in Latin; Cheiloschisis—Cleft lip; Cheilognathoschisis—Cleft lip and alveolar ridge; Cheilognathouranoschisis—Cleft lip, alveolar ridge, and palate; Uranoschisis—Cleft palate; “-” indicates concentration of interleukin in the sample was below the negative control (undetectable factor); “*” indicates the absorption level was above the OD4 level and hence, not measurable.

**Table 5:** Correlation (Spearman Rho) matrix between cytokines investigated in the present study.

Cytokines	IL-2	IL-4	IL-6	IL-10	IL-12	IL-13	IL-17A	IFN-γ	TNF-α	G-CSF	TGFβ-1
IL-2	1.000	0.700	0.515	0.771	0.600	0.231	0.842 **	0.952 **	−0.056	0.809 **	−0.071
IL-4	-	1.000	0.800	0.900 *	0.900 *	0.700	1.000 **	1.000 **	1.000 **	0.600	N.D.
IL-6	-	-	1.000	0.900 *	0.900 *	0.674 *	0.480	0.802 **	0.478	0.729 *	0.072
IL-10	-	-	-	1.000	1.000 **	−0.086	1.000 **	0.800	0.771	0.700	−1.000
IL-12	-	-	-	-	1.000	0.600	1.000 **	0.800	0.900 *	0.700	N.D.
IL-13	-	-	-	-	-	1.000	0.176	0.624	0.007	0.709 *	0.238
IL-17A	-	-	-	-	-	-	1.000	0.900 **	−0.188	0.770 **	0.893 **
IFN-γ	-	-	-	-	-	-	-	1.000	−0.067	0.903 **	0.371
TNF-α	-	-	-	-	-	-	-	-	1.000	−0.036	−0.262
G-CSF	-	-	-	-	-	-	-	-	-	1.000	0.643
TGFβ-1	-	-	-	-	-	-	-	-	-	-	1.000

* *p* value is significant at 0.05; ** *p* value is significant at 0.01; N.D.-Not Determined. Negative sign indicates a negative correlation between the cytokines.

## Data Availability

The datasets used and/or analyzed during the current study are presented in the results section of the present study.
